# Protocol for Reducing COVID-19 Transmission Risk in EEG Research

**DOI:** 10.21203/rs.3.pex-974/v2

**Published:** 2020-07-22

**Authors:** Aaron M. Simmons, Steven J. Luck

**Affiliations:** University of California, Davis

**Keywords:** EEG, electroencephalography, event-related potentials, ERPs, human neuroscience, active electrodes, COVID-19

## Abstract

Electroencephalogram (EEG) recordings provide a valuable, noninvasive methofd for measuring human brain activity. This protocol modifies our general protocol for EEG recording (Farrens et al., 2019) for use during the COVID-19 pandemic. It was created with the help of numerous experts, and it specifies a clear set of steps for interacting with research participants, using personal protective equipment (PPE), and disinfecting equipment, all with the goal of reducing the COVID-19 risks for both laboratory personnel and participants. It focuses on the use of EEG in relatively simple research studies of adults who can easily understand and follow instructions, yet can be readily adapted for studies using other types of EEG experiments or other participant populations.

## Introduction

EEG recordings provide a valuable noninvasive method for measuring human brain activity related to perception, cognition, emotion, and action. They play a major role in efforts to understand, diagnose, and treat a variety of neural and psychological conditions that cause enormous human suffering. However, EEG recordings ordinarily require close contact between an experimenter and the research participant, and they therefore create a risk of spreading the SARS-CoV-2 virus that is responsible for the COVID-19 pandemic that began in late 2019. Consequently, most EEG/ERP research was paused during the first stages of this pandemic.

A long pause in EEG/ERP research could cause a significant delay in the development of diagnostic tools and new treatments for a variety of conditions that produce significant human suffering, especially if this pause causes trainees and early career researchers to leave the field. This is true even for basic science studies of fundamental neural and psychological mechanisms that are not designed to address a specific mental or physical health condition; these studies will provide the scientific backbone for future preclinical and clinical research focused on diagnosis and treatment. The history of science shows that it is difficult to predict which basic science studies will ultimately lead to important applications, so good science is important even when it is not directed at specific applications.

Thus, it is important for researchers to resume EEG recordings as soon as the recording procedures can be made sufficiently safe for the research personnel and the research participants. Of course, researchers should not resume data collection until allowed by their institutions and the relevant government authorities, and data collection may need to ramp up and down multiple times as conditions change. However, during periods in which human subjects research can be conducted, steps should be taken to make EEG recordings as safe as reasonably possible. The goal of this protocol is to outline a set of procedures–such as wearing personal protective equipment (PPE) and disinfecting the equipment–that are designed to reduce the risks of EEG recordings in the context of the COVID-19 pandemic. Ideally, the risks should be no greater than the other risks an individual faces in daily life during the pandemic (e.g., the risks involved in grocery shopping or getting a haircut).

This protocol is designed to be used in conjunction with our general protocol for EEG recording (Farrens et al., 2019). It focuses on the use of EEG in relatively simple research studies of adults who can easily understand and follow instructions. A broader protocol that covers clinical recordings or other types of research would be unwieldy, but the present protocol could be readily adapted for studies using other types of EEG experiments or other participant populations. It could also be modified for other types of physiological or behavioral methods. We recommend that each individual investigator create their own version of this protocol that reflects the specifics of their own research environment. Indeed, we have created a simplified and more specific version of this protocol for daily use in our own lab. Related guidelines are also available for clinical neurophysiology and electrodiagnostic testing (Desai et al., 2020; San-Juan et al., 2020), and noninvasive brain stimulation (Bikson et al., 2020).

This protocol was developed using an iterative process. We first read the existing literature and discussed the general issues with individuals in our lab, with other investigators at the UC Davis Center for Mind & Brain, and with several of our collaborators at UC Davis and other institutions. We then created an initial draft and received helpful discussions and/or detailed comments from a set of basic scientists and neurologists: Emily Kappenman, George R. Mangun, David Corina, Erik St. Louis, Joshua Ewen, Jens-Max Hopf, Ariel Schoenfeld. We then revised the protocol and made the revised draft available for comments on ERPinfo.org web site. We received comments from the following individuals: John Grogan, Marc Joanisse, Joel Snyder, Emily Meachon, Ramesh Srinivasan, Kim Wise, Carlos Mugruza-Vassallo, Kimberley Whitehead, Jasna Martinovic, Graham Holt, James Rounds, Max Gattie, and Travis Baker. We integrated the suggestions of these individuals into the protocol, leading to the present version. We thank everyone who contributed to the process of creating this protocol.

Our COVID-19 risk reduction protocol makes the following assumptions:

1. EEG recordings will be obtained only when allowable by the relevant *governing bodies,* which include the researcher’s institution, ethics board / institutional review board, and local, regional, and national government. Some or all aspects of this protocol may require approval by one or more governing bodies, and a change to the informed consent documents may be necessary.
2. More restrictive rules provided by the governing bodies will always take precedence over the procedures described here. However, the procedures described here should be used even if they are more restrictive than those required by the governing bodies (which may not be designed for the specific characteristics of EEG recordings).
3. Research should remain paused until the researcher can obtain the supplies described here (e.g., personal protective equipment, disinfectants). Researchers should consult their own institutions for assistance in obtaining supplies.
4. Research subjects will not be coerced into participating in studies, and laboratory personnel will not be pressured into working in the lab.
5. The goal is not to completely eliminate all risks, because life always involves risks. Instead, the goal is to reduce the risks as much as is realistically possible, ideally to the level of risks one would experience outside of the lab.
6. Although each individual risk mitigation method described in this protocol might be insufficient in isolation, the combined use of all of these methods can significantly reduce the overall risk level.
7. Any screening procedures (e.g., health questionnaires) should be administered in a way that preserves confidentiality. For example, an online screening questionnaire should simply record whether an individual has passed or failed the screening and not the answers to any of the individual screening questions. Similarly, any laboratory personnel who do not wish to collect data will be allowed to exclude themselves without giving any reason.
8. Knowledge of how COVID-19 spreads is likely to evolve over time, and new testing and mitigation methods may become available. In addition, we will likely refine the procedures once we start using this protocol to collect data. Thus, this protocol will be updated when appropriate.

The key elements of our protocol are as follows:

1. The primary goals are to reduce the expulsion of virus into the air and to remove the virus from surfaces.
2. As much of the procedure as possible should be conducted in advance or without direct contact (e.g., consenting, preparation of electrode cap). Most importantly, the duration of the electrode application procedure should be minimized, because this involves the closest contact between participant and experimenter.
3. Both the participant and the experimenter should be screened prior to the recording session, and anyone with COVID-related symptoms or recent contact with a likely COVID-19 case should not enter the lab.
4. Both the participant and experimenter should wear appropriate personal protective equipment (PPE) at all times.
5. Anything that might be touched by the participant or experimenter (including the electrodes and cap) should be rigorously disinfected.
6. The electrode application process should be performed in a different room/area from the recording, and the experimenter should spend as little time as possible in the recording area.

Note: Some elements of this protocol may require approval from the institution’s ethics committee / institutional review board or from a grant sponsor and/or changes to the informed consent document.

## Reagents

### Equipment

Only items that have been updated or added for this protocol are included in this list; for the entire list of equipment, see Farrens et al. (2019).

#### Electrode Application Materials

1. Disposable Latex or Nitrile Gloves
  - Either nitrile or latex is effective (Rego & Roley, 1999), but nitrile is preferred for people with latex allergies. Vinyl gloves should not be used.
2. Disposable Surgical-Style Masks
  - The mask for the subject should have ear loops rather than ties to avoid interfering with the electrodes; the mask for the experimenter can have either. Do not use N95 masks; the supply is limited, and they are not necessary for this protocol. Both the participant and experimenter will be required to wear masks, greatly limiting the amount of airborne virus. Washable masks would be acceptable if they are professionally produced and of high quality (ideally meeting the *FFP1* standard); we do not recommend homemade masks. The masks must fit properly so that the individual’s breath passes through the masks rather than venting out at the edges.
3. Lab Coats or Other Coverings
  - For experimenters to wear in all subject preparation areas and EEG testing areas. These could be disposable; if not, they must be laundered at the end of each day. We will use the term “lab coat” below, but other coverings (gowns or smocks) are also acceptable as long as they cover the arms, torso, and most of the legs.
4. Face Shields
  - For experimenters to wear while close to the subject. For high-risk groups, it may be worth having the subject also wear eye protection (e.g., goggles) during the electrode application procedure.
  - Reusable face shields are preferred to reduce waste, but they must be carefully disinfected after each use.
5. Forehead Thermometer
  - We recommend avoiding no-contact (infrared) thermometers because they are typically not as reliable as ones that contact the forehead. They should be disinfected after each use. We are using the iProven DMT-489 (https://iproven.com/products/forehead-and-ear-thermometer-dmt-489-black)
6. Hand Sanitizer

#### Subject Prep and Recording Areas

Separate areas should be used for electrode application and EEG recording (to minimize the number of people who are in the recording area, where the subject will spend considerable time). If possible, the subject should be in an isolated recording room and the experimenter should be in a separate control room during the recording. If this is not possible, the experimenter and subject should be separated by at least 2 m during the recording, preferably with a barrier between them (e.g., an acrylic panel).

The subject prep area should be set up to minimize face-to-face contact between the experimenter and subject. In particular, we recommend that the subject sit at a desk or table. It may be helpful to use a chin rest to keep the subject’s head facing forward (but then this must be disinfected). To the extent possible, the experimenter will stand behind the subject while attaching the electrodes. If available, open windows may be helpful for improving ventilation.

Care should be taken to ensure proper ventilation of both the preparation area and the testing area. However, ventilation is difficult to test and control, so the main goal of the mitigation procedures should be to minimize expulsion of virus into the air.

It may be desirable to provide a bin or shelf for participants to place their personal items (e.g., purses, backpacks) during the recording procedure. This would need to be disinfected after the participants leave.

#### Clean-Up Supplies

1. Isopropyl Alcohol Wipes (>=70%)
2. Isopropyl Alcohol Solution (>=70%) or other disinfectant solution approved for use with COVID-19 (see list of EPA-approved disinfectants here)

#### Surface Coverings

All surfaces that might be touched by a subject or experimenter (e.g., response devices, door handles) must be disinfected immediately after each recording session. This is straightforward for hard (nonporous) surfaces, but difficult to do with some furniture and electronic devices (such as keyboards and gamepad response devices). Thus, all furniture and electronic devices should be fitted with waterproof covers to facilitate disinfecting procedures. For example, fabric chairs should be fitted with disposable plastic covers that will be changed after every experiment (link) or a non-disposable vinyl covering that can easily be disinfected after each subject (link). If electronic devices cannot be fitted with a covering, then spray-on disinfecting procedures can be replaced with individually packaged alcohol wipes with at least 70% isopropyl alcohol (CDC). Researchers may wish to switch from keyboards to other response devices that are more easily covered or disinfected, such as game pads and button-free mice.

#### Subject Comfort

All subject comfort items must be individually packaged (e.g., snacks, beverages) or washable (e.g., blankets). Laboratories may consider discontinuing snacks and beverages; at a minimum, hand sanitizer should be used prior to consumption.

## Procedure

### Institutional Policies

Some aspects of EEG research may be more appropriately determined and implemented by a researcher’s institution (including subunits of the institution, such as a department or building). In such cases, the institutional policies will typically take precedence over laboratory policy. We anticipate that many institutions will adopt the following policies:

#### Symptom screening

Researchers and subjects who exhibit symptoms of COVID-19 (e.g., from this CDC list) or who have had recent contact with someone with COVID-19 may not be allowed to enter the building and/or laboratory. If rapid SARS-CoV-2 testing is available, participants and researchers may be required to be tested before entering the building. Rationale: Allowing individuals with evidence of COVID-19 infection into the building or laboratory may increase the risks substantially.

#### Daily temperature screening

Researcher personnel may be asked to take their temperature once or twice per day. Rationale: This may help prevent infected persons from entering the laboratory.

#### Risk factor screening

Researchers and subjects who are over age 65 and/or have health conditions associated with serious complications of COVID-19 (e.g., as described by Zheng et al., 2020 and Lighter et al., 2020) may be precluded from participating in in-person data collection. Rationale: It is impossible to guarantee that the risk of infection from an EEG experiment will be zero, so it may be desirable to exclude individuals who are more likely to experience serious complications of an infection, especially in research with no direct benefits to the subjects or no near-term benefit to health and well-being.

#### Face masks while in public spaces

Both subjects and experimenters may be required to wear masks while in any public areas of the building (including hallways and bathrooms). Rationale: This will reduce the spread of airborne virus particles.

#### Personnel density

The number of people (including both researchers and subjects) within a laboratory area may be limited. The specific limits will depend on the nature of the space, the research, and the participants. Rationale: This will decrease the viral load and facilitate contact tracing.

#### Logging of visitors

Laboratories may be required to maintain a log of who enters a building or laboratory, the time of entry and departure, and contact information (email and phone number). Rationale: This will facilitate contact tracing.

#### Notification of infections

Subjects may be asked to contact the lab if they develop a COVID-19 infection after the session (e.g., within 14 days), and the lab may be required to contact all subjects and research staff who were in the lab within a short time (e.g., 48 hours) of an infected person. Rationale: This will facilitate contact tracing.

Note: If the institution does not have policies regarding these issues, then we recommend that individual laboratories consider adopting them.

### Modifications to General Laboratory Policies

1. Disposable masks must be worn at all times by everyone in the laboratory. A given mask may not be worn for more than one day before being discarded.
2. Experimenters should wear a lab coat and gloves whenever they are in the subject prep or testing areas.
3. No one may be in the subject prep and testing areas except the participant and 1–2 experimenters. For example, prepping two participants at the same time in the same room is prohibited.
4. Experimenters should spend as little time as possible in the testing area (during, before, and after a testing session)
5. Additional time should be scheduled between participants to allow time for cleaning/disinfecting and to ensure that one participant does not arrive before the previous participant has left.
6. Hair washing should be discontinued (unless the subject can do it alone in a separate room that will then be disinfected). Participants should be notified in advance that some gel will likely remain in their hair at the end of the session.
7. It may be appropriate to postpone experiments that require unusually prolonged periods of contact between the experimenter and the subject until the pandemic is over.

### Training

All laboratory personnel must be trained in the appropriate use of PPE, in handwashing and hand sanitizer application, and in disinfecting procedures. Experimenters must monitor research participants to ensure that their PPE fits properly when first applied, that the PPE continues to fit properly throughout the session, and that the participants wash or sanitize their hands properly.

### Before the Participant Arrives

#### Online Consent and Survey Administration

If possible, any of the ordinary screening, consent, and survey forms should be filled out online before the participant arrives. If it is not possible to use online forms, the forms should be completed by the participant in a well-ventilated and easy-to-clean space, at least 2 m from other people. At the time of consent, the participant will also be given a brief description of the risk mitigation procedures.

#### Electrode Cap Pre-Measurement (Optional)

The participant should be asked to measure themselves for cap size by giving them an infographic or guide to measure circumference of their own head. If participants do not own an appropriate measuring tape, one can be mailed to them. This allows the experimenter to set up the cap before the subject arrives, which minimizes time in the laboratory. If the cap size is not known in advance, and enough electrode sets are available, multiple caps can be prepared in the most common sizes.

#### Other Procedures Prior to Arrival

1. As usual, send a reminder email 1–2 days before the study. Add a description of any procedures required by the institution for entry into the building/laboratory (e.g., symptom screening questionnaire, waiting at the door to the building). If necessary, adjust the usual list of reminders (e.g., noting that some gel may remain in the hair at the end of the session).
2. Lay out as much of the equipment prior to the subject’s arrival as you can. This includes a gel-filled syringe, syringe tips, towels, electrode collars, gloves, and alcohol wipes. See Step 2 in *Prior to Arrival* according to Farrens et al. (2019).
3. Using measurements supplied by the participant, prepare the electrode cap for the subject’s head.
4. Everything that the subject or experimenter might touch should be disinfected (see Cleaning/Disinfecting Procedures below), even if everything was disinfected after the previous subject (unless the time between sessions is relatively short so that we can be sure that nothing was touched between sessions).

### When the Participant Arrives

1. While wearing a lab coat, gloves, and mask, meet the participant at the building entrance. Immediately have the participant put on a mask and apply hand sanitizer. The participant must wear the mask at all times while in the building, and the mask should be discarded when the participant leaves.
2. If participants arrive with their own PPE, they will be required to wear the PPE that we provide (so that we can ensure effectiveness). They may use their own hand sanitizer if it meets the typical standards for clinical use (>=60% ethanol or 70% isopropanol).
3. Any institutional policies regarding building/laboratory entry (e.g., temperature screening) should be followed (see Institutional Policies above).

### Modifications to Electrode Application Procedure

We ordinarily have subjects vigorously comb/brush their hair/scalp to reduce impedance. This step should be performed by the subject at home rather than in the lab.

Talking should be minimized, focusing on the necessary instructions and answering questions about the procedure.

The following may be appropriate to reduce the duration of the electrode application procedure and increase safety in some experiments:

- Reducing the number of electrodes
- Reducing or eliminating electrodes near mucous membranes (e.g., EOG electrodes)
- Increasing impedance thresholds

### Specific Procedures

1. Wash hands for at least 20 seconds (or apply hand sanitizer)
2. Put on a face shield, which should remain on while applying the electrodes at the beginning of the session, while making any adjustments to the electrodes during the session, and while removing them at the end of the session. The experimenter should already be wearing gloves, a lab coat, and mask.
3. Lead the participant into the subject prep area while maintaining a distance of at least 2 m. Continue to maintain this distance except when close contact is required.
4. Seat the subject in the subject prep area (optional: ask them to rest their chin on a chin rest affixed to the prep table to keep them from facing the experimenter).
5. If the subject has not already provided head measurements, measure the circumference of the subject’s head using a disposable soft tape measure, select the appropriate cap, and attach the electrodes.
6. Continue with the rest of the electrode application procedure, as described in Farrens et al. (2019).
7. Wash hands again for at least 20 seconds (or apply hand sanitizer)
8. Note: the subject should continue to wear a mask throughout this set of procedures. The mask should minimally interfere with electrodes and cap placement. It also should not produce any artifacts.

### Modifications to the Data Collection Procedure

Execute steps 1–17 of *Running the Subject* according to Farrens et al. (2019), but maintain a distance of at least 2 m between participant and experimenter as much as possible. For example, whereas the experimenter would normally be in the recording area with the subject during the artifacts demo and task instructions, the experimenter should instead perform these steps via the intercom system. However, the experimenter may need to enter the recording area briefly (e.g., to connect the subject to the amplifier, to adjust any problematic electrodes).

To keep the control area clean, experimenters should wash their hands or apply hand sanitizer after entering the recording area or having any physical interaction with the subject.

Reminder: Both the experimenter and the subject must wear masks during the entire data collection period (even if a closed door separates the control area from the recording area). The experimenter should continue wearing a lab coat but need not wear gloves except when in the recording area.

It may be desirable to have the subject wear gloves during the data collection period to minimize surface-to-face transfer of the virus. However, this would likely be uncomfortable for experiments lasting more than ~15 minutes. Also, the latest advice (as of May 22, 2020) from the CDC is that transmission from surfaces is relatively rare, but this is subject to change.

### Modifications to the Cleaning/Disinfecting Procedures

Execute steps 1–16 of *Clean Up* according to Farrens et al. (2019), immediately upon the subject’s departure, with the following changes:

- The experimenter will wear a lab coat, gloves, and a mask throughout these procedures.
- Participant hair washing is discontinued (unless the subject can do it alone in a separate room that will then be disinfected). Instead, gel should simply be wiped from the hair to the extent possible with a tissue or gauze pad.
- After the electrodes have been removed and the gel is wiped from the subject’s hair, the subject should be walked to the exit, then asked to apply hand sanitizer, and finally asked to remove and discard the face mask. The experimenter should then apply hand sanitizer. A hand sanitizer dispenser and trash bin should be placed by the building exit to facilitate these steps.
- Step 9 & Step 11: Disinfect the electrodes and electrode caps in Envirocide solution for 3 minutes.
- Although Envirocide has not been tested with SARS-CoV-2, it is active against enveloped viruses such as the Coronavirus family. Indeed, enveloped viruses (such as those in the Coronavirus family) are the easiest to kill.
- This disinfecting procedure is recommended for the Brain Products actiCAP system. If you are using a different system, contact the manufacturer for disinfecting advice.
- As usual, used syringes and tips are discarded.
- Discard or disinfect plastic chair covers.
- Using a 70% isopropyl solution or other approved disinfectant, spray and wipe down:
- All chairs and table surfaces in both the preparation area and testing area
- All response devices
- All high-touch surfaces such as door handles
- Anything that might be touched by the subject or the experimenter (e.g., the microphone for the intercom system)
- The experimenter’s gloves can be discarded and the lab coat can be removed once the subject has departed.
- The lab coat must be washed at the end of the day before it can be worn again.
- The experimenter’s mask should be discarded and hands should be sanitized when the experimenter leaves the building.

## Troubleshooting

### Time Taken

Set-up Prior to Participant Arrival (including participant correspondence, consenting, electrode cap prefitting, PPE, etc): 45 mins

Electrode application: 5–15 mins (for 32 electrodes)

Impedance adjustments: 5–10 mins

Recording: variable (depending on task duration)

Electrode removal and clean up: 20–30 mins

### Anticipated Results

These procedures were designed to help reduce the COVID-19 risks for both laboratory personnel and participants during the current pandemic while maintaining high EEG recording standards.
